# Supplementary material for: Spatially selective manipulation of cells with single-beam acoustical tweezers
Source: Nat Commun. 2020 Aug 25;11:4244. doi: 10.1038/s41467-020-18000-y (PMC7447757; doi:10.1038/s41467-020-18000-y)
Supplement: Supplementary file 2 — Supplementary Information [file 41467_2020_18000_MOESM2_ESM.pdf]

Spatially selective manipulation of cells with single-beam acoustical tweezers: Supplementary note

Michael Baudoin,[1,2,*] Jean-Louis Thomas,[3] Roudy Al Sahely,[1] Jean-Claude Gerbedoen,[1] Zhixiong Gong,[1] Aude Sivery,[1] Olivier Bou Matar,[1] Nikolay Smagin,[1] Peter Favreau,[1] and Alexis Vlandas[1,†]

[1] *Univ. Lille, CNRS, Centrale Lille, Yncréa ISEN, Univ. Polytechnique Hauts-de-France, UMR 8520 - IEMN, SATT NORD, F- 59000 Lille, France.*

[2] *Institut Universitaire de France, 1 rue Descartes, 75005 Paris*

[3] *Sorbonne Université, CNRS, Institut des NanoSciences de Paris, INSP, F-75005 Paris, France*

(Dated: July 16, 2020)

* Corresponding author: michael.baudoin@univ-lille.fr; http://films-lab.univ-lille1.fr/michael

† Corresponding author: alexis.vlandas@iemn.fr

I. POSSIBILITY AND LIMITS OF VORTEX-BASED TWEEZERS

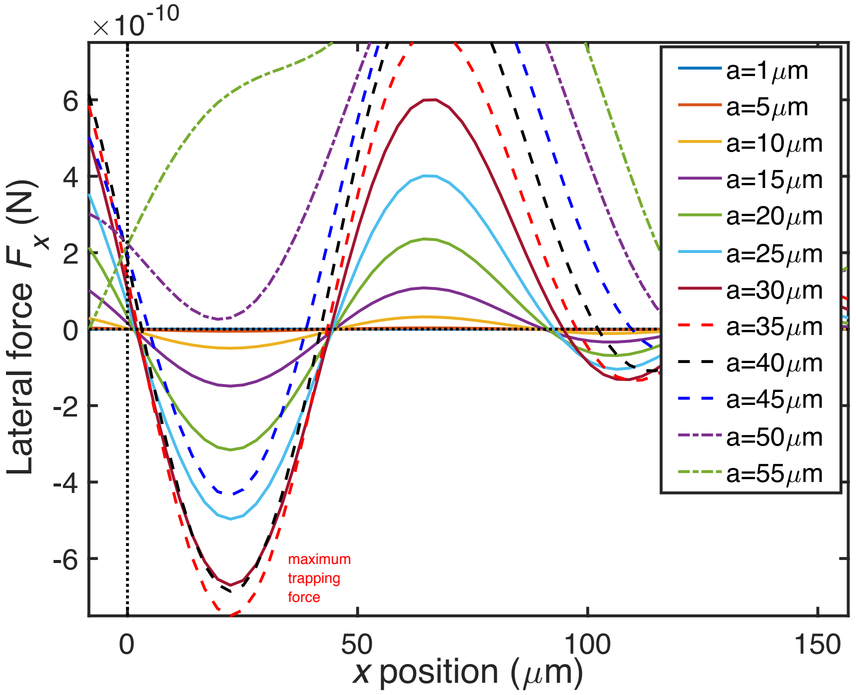

FIG. 1. Lateral force F_x exerted on a cell, as a function of the cell position x for different cell radii a for the acoustical beam represented on Fig. 2.A, an acoustic maximal normal displacement (magnitude) of 1 nm and cells density and compressibility of 1100 kg m^{-3} and 4×10^{-10} Pa^{-1} respectively

In this section, we study numerically how the lateral trapping force evolves as a function of the size of the trapped cell (see Fig 1). The incident signal here is the one of tweezers 2 producing the field represented on Fig. 2 at an actuation frequency of 43.5 MHz. This figures shows that all cells of radius $a \leq 45$ µm are attracted toward the center of the vortex (the force is negative), while larger particle are expelled. The maximum trapping force is obtained for cells of radius $a =$35 µm. These two distances are comparable with the radius of the first ring (distance to the maximum amplitude of vibration), which according to Fig. 2, is ~ 40 µm. Hence the maximum trapping force is obtained when the radius of the particle matches with the lateral extension of the first ring. This also corresponds to the optimal selectivity, since in this case the spatial selectivity would match the radius of the

trapped object. Since the radial variation of the wavefield can be described with a spherical Bessel function $j_1(kr)$ [1], this optimal force and selectivity criterion can be summarised as $d/\lambda \sim 0.7$. with d the cell diameter and λ the field wavelength. Hence for 2D trapping, the trapping force and selectivity could be further increased by designing higher frequency tweezers. Indeed, based on considerations on the attenuation length in water, one can envision to manipulate cells in a channel with a height corresponding to a few diameter of the trapped object up to frequencies of several hundred MHz, with the sound absorption in water limited to less than 10% of the incident wave. Nevertheless, other source of dissipation (Joule heating in the electrodes, glue, ...) should be also carefully managed. In addition, for 3D trapping, an axial trap is obtained only if the radius of the particle is significantly smaller than the radius of the first ring (typically $d/\lambda \sim 0.3$ [2]). Otherwise the particle is expelled axially from the vortex center. The present tweezers were built with this constraint in mind.

[1] M. Baudoin, J.-C. Gerbedoen, A. Riaud, O. Bou Matar, N. Smagin, and J.-L. Thomas, "Folding a focalized acoustical vortex on a flat holographic transducer: miniaturized selective acoustical tweezers," Science Adv. **5**, eaav1967 (2019).

[2] D. Baresch, J.-L. Thomas, and R. Marchiano, "Observation of a single-beam gradient force acoustical trap for elastic particles: acoustical tweezers," Phys. Rev. Lett. **116**, 024301 (2016).
