# Supplementary material for: Spatially selective manipulation of cells with single-beam acoustical tweezers
Source: Nat Commun. 2020 Aug 25;11:4244. doi: 10.1038/s41467-020-18000-y (PMC7447757; doi:10.1038/s41467-020-18000-y)
Supplement: Supplementary file 10 — Description of Additional Supplementary Files [file 41467_2020_18000_MOESM10_ESM.pdf]

Title: Supplementary Movie 1
Description: Movie illustrating the working principle of acoustical tweezers based on focused acoustical vortices.

Title: Supplementary Movie 2
Description: Movie showing the selective displacement of a MDA-MB-231 cell of radius 7 ± 1 µm between other free cells. Then, one of the free cells initially serving as a slalom pole is moved in turn to demonstrate that it was free to move. Movie accelerated by a factor 2. Manipulation was performed with Tweezer 1.

Title: Supplementary movie 3
Description: Movie showing the precise selective displacement of a MDA-MB231 cell of radius 7 ± 1 µm along a square and around another cell. Movie accelerated by a factor 2 obtained with Tweezer 2.

Title: Supplementary Movie 4
Description: Movie showing (i) the influence of the repulsive ring surrounding the trapped cells on other neighboring cells and (ii) how the repulsive ring can be used to pick up. The manipulated cells are some MDA-MB-231 and manipulation was performed with Tweezer 1.

Title: Supplementary Movie 5
Description: Moving showing the manipulation of a MDA-MB-231 cell in between other cells when there is a slight adherence of the cells on the substrate. The acoustic power is increased to detach the cell and then reduced for the displacement sequence. Manipulation was performed with Tweezer 1.

Title: Supplementary Movie 6
Description: Movie showing rapid displacements of a MDA-MB-231 cell. Real time. First part of the movie: a cell is moved at an average speed of 0.4 mm s^{-1} with a driving acoustic displacement magnitude of 0.5 nm (Tweezer 3). Second part of the movie: a cell is moved up to a maximum speed of 1.2mm s^{-1} with an acoustic magnitude of 0.9 nm (Tweezer 1).

Title: Supplementary software 1
Description: Angular spectrum code simulating the propagation of the focused acoustical vortex from the source to the focal plane and the lateral force field in the focal plane.
